# The Role of Interferon in the Management of BCG Refractory Nonmuscle Invasive Bladder Cancer

**DOI:** 10.1155/2015/656918

**Published:** 2015-10-13

**Authors:** Andres F. Correa, Katherine Theisen, Matthew Ferroni, Jodi K. Maranchie, Ronald Hrebinko, Benjamin J. Davies, Jeffrey R. Gingrich

**Affiliations:** Department of Urology, University of Pittsburgh Medical Center, 3471 5th Avenue, Suite 700 Kaufmann Building, Pittsburgh, PA 15213, USA

## Abstract

*Background*. Thirty to forty percent of patients with high grade nonmuscle invasive bladder cancer (NMIBC) fail to respond to intravesical therapy with bacillus Calmette-Guerin (BCG). Interferon-*α*2B plus BCG has been shown to be effective in a subset of patients with NMIBC BCG refractory disease. Here we present a contemporary series on the effectiveness and safety of intravesical BCG plus interferon-*α*2B therapy in patients with BCG refractory NMIBC. *Methods*. From January of 2005 to April of 2014 we retrospectively found 44 patients who underwent induction with combination IFN/BCG for the management of BCG refractory NMIBC. A chart review was performed to assess initial pathological stage/grade, pathological stage/grade at the time of induction, time to IFN/BCG failure, pathological stage/grade at failure, postfailure therapy, and current disease state. *Results*. Of the 44 patients who met criteria for the analysis. High risk disease was found in 88.6% of patients at induction. The 12-month and 24-month recurrence-free survival were 38.6% and 18.2%, respectively. 25 (56.8%) ultimately had disease recurrence. Radical cystectomy was performed in 16 (36.4%) patients. *Conclusion*. Combination BCG plus interferon-*α*2B remains a reasonably safe alternative treatment for select patients with BCG refractory disease prior to proceeding to radical cystectomy.

## 1. Introduction

In 2014, approximately 75,000 new cases of bladder cancer will be diagnosed in the USA [1]. At diagnosis, roughly 80% of bladder tumors will be classified as nonmuscle invasive. Nonmuscle invasive bladder cancer is prone to recur and, worse, progress to muscle invasive disease. Bacillus Calmette-Guerin (BCG) is the only intravesical agent proven to reduce rates of recurrence and to delay progression in intermediate and high risk nonmuscle invasive bladder cancer (NMIBC) [2]. The approximately 30% to 40% of patients who will fail BCG therapy represent a frequent dilemma to the treating physician. Although 50% of patients who did not respond to initial therapy will respond to a second induction regimen [3], failure of reinduction with BCG is associated with recurrence and progression with a 30% chance of developing muscle invasive disease [4]. Early radical cystectomy has been shown to improve survival in this patient population [5–7]; however, it carries a significant degree of morbidity, mortality, and life-style changes. Sadly, there remains no reliable gold standard salvage intravesical therapy for this cohort of patients.

While the etiology of BCG failure remains unclear, there are various factors that may explain this phenomenon. Intravesical BCG acts as an immune modulator which elicits a TH1-type response in the bladder. When an appropriate TH1-type response is triggered, chemokine signaling elicits the recruitment of monocytic and granulocytic lymphocytes capable of eliminating bladder tumor cells [8]. Failure to elicit this immune response leads to BCG failure. This occurs when an insufficient BCG concentration is used or a predominant TH2-type immune response is elicited.

Interferon-*α* (IFN-*α*) is a pleiotropic immune modulator that has demonstrated antiproliferative activity in several preclinical studies. While the results of interferon-*α*2*β* (IFN-*α*2*β*) as intravesical monotherapy has proven to be inferior to standard therapies [9],* in vitro* studies have shown that the addition of IFN-*α*2*β* to BCG potentiates the TH1-type response [10]. O'Donnell and colleagues proposed the addition of IFN-*α*2*β* to a BCG regimen with the hope it would synergistically elicit an appropriate host response in patients who have failed induction BCG therapy. In the original study consisting of 40 patients, a good response to combination therapy was observed with 12-month and 24-month disease-free rates of 63% and 53%, respectively [10]. This data was further explored in a multicenter randomized trial by Joudi et al. [11] which included 1,007 patients and reported a lower 2-year disease-free rate of only 45% after combination therapy in patients who had failed BCG induction therapy. Consequently, newer salvage intravesical treatments with chemoagents, thermochemotherapy, and electromotive therapy have been introduced to try to improve on these results for patients following BCG failure.

Herein we review our single institution contemporary experience of combination BCG plus IFN-*α*2*β* as salvage intravesical therapy for patients with NMIBC who failed BCG therapy and, secondly, perform a literature review of the newly introduced salvage intravesical therapies to place in perspective the current role of IFN-*α*2*β* in the salvage treatment of BCG refractory NMIBC.

## 2. Methods

We retrospectively reviewed the charts of patients who underwent treatment with combination therapy, BCG plus IFN-*α*2*β* for the treatment of BCG refractory NMIBC from January of 2005 to April of 2014. BCG refractory status was defined as worsening or nonimproving disease despite full induction or maintenance course of BCG therapy. All patients were treated per O'Donnell et al.'s intravesical protocol which constitutes 6 weekly installations of 1/3 BCG dose plus 50 million units of interferon-*α*2*β* diluted in 50 cc of buffered saline [12]. If induction was successful patients were continued in a maintenance protocol with instillations at 3, 6, 12, 18, 24, and 30 months, respectively. Patients were surveyed at 3-month intervals during the 1st year, 6 months during the 2nd year, and annually thereafter. Failure was determined when a bladder recurrence was noted during the surveillance period. Patients that failed were again offered a bladder extirpation procedure. BCG naïve or intolerant patients along with patients presenting upper tract disease who received combination therapy were excluded from the analysis.

Charts were reviewed to assess initial pathological stage/grade, pathological stage/grade at the time of induction, time to IFN/BCG failure, pathological stage/grade at failure, postfailure therapy, and current disease state. Pearson chi-square tests were performed to analyze patient and/or tumor characteristics associated with failure of combination therapy. Analyses were performed using SigmaXL software (SigmaXL, Toronto, Ontario, Canada) with *p* values < 0.05 being considered statistically significant.

## 3. Results

The initial search revealed 50 patients who underwent intravesical combination therapy with BCG plus INF-*α*2*β* for treatment of urothelial carcinoma, though 4 patients were noted to be BCG naïve and 2 were found to have an upper tract disease and therefore excluded. Therefore, 44 patients met inclusion criteria for analysis of which 35 (79%) were male, as shown in Table 1. Thirty-one (70%) patients underwent combination therapy with the goal of bladder preservation rather than cystectomy. The remainder 13 (30%) patients had severe comorbidities prohibiting radical cystectomy. Median age at time of diagnosis was 63 years (38–92). The median ASA class for the overall cohort was 3 (2–4), while the ASA class for the bladder sparing group was 2 (2-3). The most common stage at induction was pTa (50%) followed by pT1 (45.5%), with 88.6% of tumors displaying high grade disease. Patients who had failed BCG within 6 months were common, accounting for 43% of the entire cohort. Of the patients that failed BCG within 6 months, 9 (47.3%) failed within 3 months and 16 (89.4%) received a second BCG induction prior to combination therapy. All patients but 7 (16%) patients tolerated induction therapy with 28 (63.6%) patients continuing on maintenance therapy. Six (14%) patients did require treatment for a UTI during the induction phase. One patient developed a postinstallation fever requiring admission and treatment with antituberculin agent.

Of the 44 patients, 19 (43.2%) were recurrence-free with median follow-up of 28 months. However, 12-month and 24-month recurrence-free rates for the cohort were only 38.6% and 18.2%, respectively. Sixteen (36.3%) patients underwent salvage cystectomy following failure. Two (4.5%) patients developed metastatic disease and there were 2 (4.5%) cancer specific deaths. The bladder preservation rate in the cohort was 61.3%, with 12-month and 24-month disease-free rates for the cohort of 86.4% and 61.4%, respectively.

A comparison of the clinical and pathological patient characteristics between failures and nonfailures is shown in Table 2. Early BCG monotherapy failure (<6 months) was significantly associated with failure of combination therapy. Larger tumors and multifocal disease were more frequent in the failure group but this difference was not found to be statistically significant.

Twenty-five (56.8%) patients experienced a recurrence at follow-up. Median time to recurrence was 7.2 months. The most common stages at recurrence were pTa and pT1 diseases which accounted for 40% and 44% tumors resected at failure. High grade disease was seen in 92% of recurrences. The incidence of upstaging and upgrading in the failure group was 36% and 12%, respectively. Three (12.0%) patients were found to have muscle invasive disease at time of recurrence. Sixteen patients underwent salvage cystectomy. Of the remaining 9 patients that refused radical cystectomy, 7 underwent salvage intravesical therapy (1 repeat BCG, 4 MMC, 1 additional BCG/INF, and 1 enrolled in a clinical trial utilizing investigational Mycobacteria cell wall DNA complex), 1 was managed with systemic therapy, and 1 refused further treatment.

At radical cystectomy 8 (50.0%) patients were found to have pT1 disease. One patient was found to have pT0 disease. Advanced stage was found in 5 (20%) patients. Of these, four presented with pT2 disease and one with pT3 disease. Micropapillary features were seen in 3 (60%) specimens showing advance stage. Four of the five patients who showed progression to an advanced stage at cystectomy had failed BCG monotherapy within 6 months. Positive lymph nodes were found in 2 (13.3%) of the 16 patients that underwent radical cystectomy. Of the patients treated with salvage cystectomy, 2 (8%) patients went on to develop metastatic disease.

At a median follow-up of 19 months following cystectomy, 5 (31%) developed postcystectomy related complications. Two patients developed hernias (1 incisional and 1 parastomal), 1 developed recurrent pyelonephritis, 1 developed left ureteroenteric anastomotic stricture, and 1 developed a urethrovesical anastomotic contracture following orthotopic neobladder diversion.

## 4. Discussion

Intravesical immunotherapy with BCG has been shown to be the most effective treatment for high risk NMIBC with response rates in order of 55% to 65% [20, 21]. Consequently, 30% to 40% of patients with high grade NMIBC will ultimately fail BCG therapy [8]. Recently, there have been several reports advocating for early cystectomy due to higher risk of progression to advance disease and improve cancer specific survival in this subgroup of patients [5, 7]. Early cystectomy is not an option in a significant portion of this population due to patient's unwillingness to undergo major surgery, and there is potential for significant morbidity and mortality due to comorbidity competing risks. To date there is no current gold standard intravesical salvage therapy for this patient subgroup. The goal of this study is to assess the disease recurrence rate following combination therapy at our institution and to determine a population in which combination therapy would be beneficial.

At our institution, the standard of care is to offer radical cystectomy to patients with BCG refractory or resistant disease. Combination therapy with BCG plus interferon is offered to those patients who desire continued bladder preservation strategies or/and to patients that have severe medical comorbidities in whom radical cystectomy is prohibited. The cohort for this study was made mainly of patients wishing for a bladder preservation protocol with a median age of 62 years and ASA class of 2. All salvage cystectomies were performed in the bladder preservation cohort. The postcystectomy complication rate was 31% consistent with that reported in the literature [13, 22]. Patients that underwent intravesical combination therapy tolerated the regimen well with 84% completing an induction phase and 64% continuing with maintenance therapy. The likely reason for this high tolerance was the exclusion of patients intolerant to BCG from the analysis and the BCG dose reduction to 1/3 of total dose.

O'Donnell and colleagues presented the initial report on the use of combination BCG plus interferon for patients who had failed BCG monotherapy. The results were encouraging with 12- and 24-month disease-free survival of 63% and 53%, respectively [12]. A multicenter randomized trial followed, consisting of 1007 patients with included BCG naïve and BCG refractory disease treated with combination therapy. At 24-month median follow-up, 59% and 45% remained recurrence-free in the BCG naïve and BCG refractory groups, respectively [11]. Lamm and colleagues also reported their experience in a series of 32 patients of which 20 had failed prior BCG therapy. At median follow-up of 22 months the disease-free survival was calculated at 50% [23].

Our series presents even lower 12-month and 24-month recurrence-free survival rates of 38.6% and 18.2. Comparing tumor pathological characteristics at induction the current series presents with a higher number of high risk malignancies with 88.6% of tumors harboring high grade features (T1, Tis, and grade 3) compared to 78% and 44% in the aforementioned series. Progression to advanced stage disease (pT2 or greater) was seen in 5 (6.8%) patients, which is comparable to the reports by O'Donnell and Lam et al. [12, 14] where the reported incidence ranged from 3% to 12.5%. A concerning finding was the presence of micropapillary features in roughly 20% of patients who underwent salvage cystectomy. At median follow-up of 28 months the bladder preservation rate was 61.3% comparable to the literature ranging from 55% to 75% [12, 23].

Our results are in line with those reported by Rosevear and colleagues, [15] where failure of induction BCG monotherapy within 6 months was associated with failure of combination BCG plus interferon therapy. Not only was BCG failure <6 months associated with failure of combination therapy but also 73.3% of patients undergoing cystectomy had failed BGC monotherapy within 6 months as well. 66.7% of the patients who developed disease progression or advanced disease were again noted to fail BCG monotherapy within 6 months. While patients with large tumors and multifocal disease at induction were more common in the failure group a statistically significant association was not seen. Timing of initial induction BCG monotherapy failure appears to be a significant predictor of salvage combination therapy failure and disease progression. Consequently, patients who fail BCG within 6 months should be even more strongly counseled towards early radical cystectomy.

Valrubicin was approved by the FDA in 1998 following a phase III study [16] for patients with BCG refractory carcinoma* in situ* (CIS). In the pivotal trial the disease-free status at 12 months was 10%, inferior to the results reported by O'Donnell and colleagues with combination BCG plus interferon. This low event-free survival (EFS) was recently validated in a retrospective study by Cookson et al. [17], where the 12-month EFS following valrubicin instillation was calculated at 16.4%. Valrubicin was seldom used during the current study period due to the low number of patients with CIS in the recurrence specimen and the fact that the medication was off the market for half of the study period (2004–2009).

Over the last 10 years several novel intravesical therapies have been proposed for the management of BCG refractory NMIBC. These can be categorized as chemotherapy, immunotherapy, and device assisted therapy. A direct visual comparison between the different treatment modalities can be seen in Table 3.

Gemcitabine is a nucleoside analogue that causes defective DNA replication, leading to tumor cell apoptosis. In a phase II trial Dalbagni et al. [18] followed up 30 BCG failure patients after administering 2 cycles of intravesical gemcitabine and cisplatin (2000 mg/100 mL) for 3 consecutive weeks. At a median follow-up of 19 months the initial CR was 50% with 12-month recurrence-free rate of 21%. Most recently Mohanty et al. [19] treated 35 patients, following BCG failure, with 2000 mg of intravesical gemcitabine weekly for 6 weeks. At median follow-up of 18 months, 60% showed no recurrence, 31% recurred with similar stage/grade, and 9% progressed to muscle invasive disease. Di Lorenzo et al. [24] randomized 80 high risk patients who had failed initial treatment with BCG to gemcitabine (2000 mg/50 mL) or a BCG (81 mg) group. At median follow-up of 15 months 52.5% and 87.5% of the patients experienced a recurrence in the gemcitabine and BCG groups, respectively. Twenty-one (33%) and 13 (37.5%) suffered disease progression requiring radical cystectomy.

Docetaxel is a semisynthetic microtubule inhibitor. Barlow and colleagues [25] treated 33 patients with BCG refractory disease with a 6-week induction course of docetaxel. The median follow-up was 20 months and the 12-month and 24-month recurrence-free survival was calculated at 45% and 32%, respectively.

Thermochemotherapy (TC) including the Synergo system incorporates a combination of intravesical mitomycin-C (MMC) and bladder wall hyperthermia using thermocouple catheter and microwave equipment. The technology is based on the finding that inducing bladder wall to temperatures of 42°C improves the absorption of sequentially administered intravesical MMC. Nativ et al. [26] reported the results of 110 patients with BCG refractory high risk NMIBC using the Synergo system. The protocol consisted of weekly TC therapy for 6–8 weeks followed by six sessions every 6–8 weeks. The reported 12-month and 24-month disease-free survival was 85% and 56%, respectively.

Finally electromotive drug administration (EMDA) has shown promise in the treatment of high risk NMIBC. The concept behind this technology is to create a current gradient between the intravesical chemotherapy agent and the bladder wall; in order, to improve the transmembrane transport of the chemotherapeutic agent. Di Stasi and colleagues [27] performed a prospective trial in 108 BCG naïve patients randomizing them to EMDA + MMC versus passive MMC versus standard BCG. Complete response rates at 3 months and 6 months were 53%, 28%, and 56% and 58%, 31%, and 64% for the EMDA + MCC, passive MMC, and standard BCG groups, respectively. Median time to recurrence was 35, 19.5, and 26 months, respectively. The authors concluded that EMDA + MMC is comparable to standard BCG therapy in patients with high risk NMIBC.

As shown in Table 3 all proposed intravesical salvage treatments for BCG failure NMIBC have similar recurrence and progression rates and the differences mainly accounted for by the different patient populations. At the moment, combination immunotherapy of BCG plus interferon has the largest volume of data for its use in this cohort of patients. While some chemotherapy and device assisted intravesical therapies show promise in small cohort studies, the institution of these therapies requires further investigation and may require investment into technology with limited in-clinic use.

## 5. Conclusion

Herein, we present our contemporary experience with combination BCG plus interferon-*α*2*β* as salvage intravesical therapy for patients with BCG refractory NMIBC. BCG plus interferon therapy appears to be effective in a subset of patients which needs to be clarified through further investigation. It is an overall well-tolerated therapy with acceptable recurrence- and progression-free rates compared to other salvage regimens. While no standardized criteria and regimen have been established for the management of this patient population, results from this study confirm, as prior series suggested, that salvage intravesical therapy should not be offered to patients who fail BCG induction therapy within 6 months.

## Figures and Tables

**Table 1:** Patient and tumor characteristics at the time of BCG/IFN induction.

Number of patients	44	
Median age (range)	63.5	(38–92)
Male	35	79.5%
Female	7	20.5%
Median ASA	3	(2–4)
Median # of BCG inductions	1	(0–10)
<2 BCG	20	45.5%
BCG = 2	9	20.5%
>2 BCG	13	29.5%
Time to BCG failure		
<6 months	20	45.5%
6–12 months	12	27.3%
12–24 months	6	13.6%
>24 months	10	22.7%
Pathology at induction		
pTis	15	34.1%
pTa	16	36.4%
pT1	13	29.5%
Grade at induction		
LG	5	11.4%
HG	39	88.6%
Failure of combination INF/BCG		
Yes	25	56.8%
No	19	43.2%
Recurrence-free at 12 months	17	38.6%
Recurrence-free at 24 months	8	18.2%
Radical cystectomy	16	36.4%
Disease-free at 12 months	38	86.4%
Disease-free at 24 months	27	61.4%
Metastatic disease	2	4.5%
Deceased at follow-up	2	4.5%
Median follow-up	28.47	(5.3–115.3)

**Table 2:** Patient and pathological tumor characteristics between BCG/IFN failures and nonfailures.

	Failures		Nonfailures		*p* value
No	25	56.8%	19	43.2%	
Male	18	40.9%	17	38.6%	0.15
Female	7	15.9%	2	4.5%	0.15
Time to BCG failure					
<6 months	16	36.4%	4	9.1%	0.0046
6–12 months	6	13.6%	6	13.6%	0.5
12–24 months	1	2.3%	5	11.4%	0.84
>24 months	6	13.6%	4	9.1%	0.21
Pathology at induction					
Tis	7	15.9%	8	18.2%	0.33
Ta	10	22.7%	5	11.4%	0.27
T1	8	18.2%	6	13.6%	0.44
Grade at induction					
HG	22	50.0%	17	38.6%	0.93
LG	3	6.8%	2	4.5%	0.93
Tumor size					
<1 cm	7	15.9%	6	13.6%	0.79
1–5 cm	16	36.4%	12	27.3%	0.95
>5 cm	2	4.5%	1	2.3%	0.72
Multifocality					
Yes	11	25.0%	6	13.6%	0.4
Hx smoking	16	36.4%	12	27.3%	0.95

**Table 3:** Studies of intravesical treatments used in patients with bacillus Calmette-Guerin failure.

Study	Treatment modality	*n*	Follow-up	Recurrence-free survival	Progression, %	Cystectomy rate, %	High risk^*∗*^ disease, %
UMPC series	BCG plus IFN-*α*2*β*	44	28 months	39% and 18% at 12 months and 24 months	12	36	86

O'Donnell et al. [12]	BCG plus IFN-*α*2*β*	40	30 months	63% and 53% at 12 months and 24 months	12	55	78

Stein et al. [13]	BCG plus IFN-*α*2*β*	32	22 months	53% at median follow-up	16	22	44

Joudi et al. [11]	BCG plus IFN-*α*2*β*	1,007	24 months	45% at 24 months	—	—	70

Lam et al. [14]	IV gemcitabine	30	19 months	21% at median follow-up	3.5	37	100

Rosevear et al. [15]	IV gemcitabine	35	18 months	60% at median follow-up	8.75	—	62

Dinney et al. [16]	IV gemcitabine	80	15.5 months	19% at median follow-up	33	33	87

Cookson et al. [17]	IV docetaxel	33	29 months	32–45% at median follow-up	—	—	76

Dalbagni et al. [18]	Thermochemotherapy	111	16 months	85% and 56% at 12 months and 24 months	3	—	26

Mohanty et al. [19]	Electromotive	108	6 months	CR 53% and 58% at 3 months and 6 months	—	—	100

^*∗*^High risk: CIS, T1, or grade ≥3.
